# Response characteristics of highland barley under freeze-thaw, drought and artemisinin stresses

**DOI:** 10.1186/s12870-022-03520-0

**Published:** 2022-03-18

**Authors:** Huichen Liu, Guozhang Bao, Zihao Dou, Haoyuan Liu, Jingqi Bai, Yingyi Chen, Yifu Yuan, Xin Zhang, Jinghui Xi

**Affiliations:** 1 College of New Energy and Environment, Jilin University, Changchun, 130012 China; 2 Key Laboratory of Groundwater Resources and Environment of the Ministry of Education, Jilin University, Changchun, China; 3 Jilin Provincial Key Laboratory of Water Resources and Environment, Changchun, China; 4 College of Computer Science&Technology, Jilin University, Changchun, 130012 China; 5 College of Biological and Agricultural Engineering, Jilin University, Changchun, 130012 China; 6 College of Plant Science, Jilin University, Changchun, 130062 China

**Keywords:** Highland barley, Freeze-thaw, Drought, Artemisinin

## Abstract

The freeze-thaw of early spring in China’s Qinghai-Tibet Plateau is often accompanied by severe droughts. *Artemisia annua*, widely distributed in China, releases allelopathic substances, mainly artemisinin, to the environment and exerts a wide range of effects on crops. This paper studied the physiological effects of highland barley under freeze-thaw, drought, and artemisinin stress through indoor simulation experiments. The physiological response characteristics of superoxide dismutase (SOD) activity, catalase (POD) activity, net photosynthetic rate, relative water content (RWC), relative electrical conductivity, malondialdehyde (MDA) content, and soluble protein content in highland barley were analyzed. The results showed that artemisinin and drought contributed to the increase of SOD activity and the decrease of POD activity. Under the freeze-thaw stress, the SOD and POD activities both decreased firstly and then increased, but the effect of compound stress on POD was more complicated. Either artemisinin, drought, or low temperature could reduce the net photosynthetic rate of highland barley. Low temperature had more significant impacts on photosynthesis, and compound stress would show a single stress superimposed effect. Artemisinin, drought, and low temperature could reduce the RWC of highland barley, and increase the relative electrical conductivity and the concentration of soluble protein (except for low temperature stress above zero, which reduces the concentration of soluble protein). However, the effect of compound stress on soluble protein is more complex. The single stress of artemisinin and drought had no obvious effect on MDA content, while the MDA content was increased significantly under the freeze-thaw stress and the compound stress of artemisinin and drought, and the MDA content reached its peak at T1. The results are helpful to explore the effects of freeze-thaw, drought and artemisinin stress on the growth of highland barley under the background of the aridification of the Qinghai-Tibet Plateau, and provide ideas for rational agricultural management.

## Introduction

Highland barley (*Hordeum distichon* L.) is one of the most important plateau cereal crops on the Qinghai-Tibet Plateau, which is the highest altitude plateau in the world as well as the largest in China [1]. In the Qinghai-Tibet Plateau, low temperature is the major limiting factor, and the freeze-thaw that is common in early spring and early winters accompanied by low temperature will also have adverse effects on crops [2]. Freeze-thaw refers to a physical geological effect and phenomenon in which the soil layer freezes and thaws owing to the temperature falling below zero and rising above zero. It mainly occurs in high latitudes and high altitude areas [3], especially early winter and early spring on the Qinghai-Tibet Plateau. Freeze-thaw not only has effects on the physiological processes of plants [4], but also destroys the structure of the soil and changes the hydrological characteristics of the soil [5], thereby affecting the yield of crops and causing huge economic losses [6].

Drought cannot be ignored because of the low precipitation as well as the large evaporation in the Qinghai-Tibet area [7]. Drought can restrain plant growth as well as development and reduce crop yields through affecting plant photosynthesis, respiration and nitrogen metabolism [8, 9].

Allelopathy, which is a chemical ecological defense mechan’ism, has favorable or unfavorable effects on the growth or development of plants by releasing chemical compounds [10]. As one of the important metabolites of *Artemisia annua*, artemisinin has attracted much attention due to its excellent anti-malarial effects [11]. *Artemisia annua*, distributed throughout China, releases allelopathic substances which is mainly composed of artemisinin into the environment [12]. On the other hand, artemisinin is widely used in medicine to produce indirect ecological effects [13–15]. However, the mechanism of artemisinin stress on the growth of barley is still unclear. Studying the physiological response of highland barley to artemisinin is helpful to deepen people’s understanding of the allelopathy of artemisinin and reduce the loss caused by the allelopathy of artemisinin.

So far, relevant research has mainly focused on the effects of drought and low temperature stress [16–19], artemisinin stress [20], or the compound stress of low temperature and drought [21, 22] on plants. There are relatively few studies on the compound stress of artemisinin. Our research placed emphasis on the effects of freeze-thaw, drought, and artemisinin stress on the physiological conditions of highland barley, providing theoretical basis and data support for guiding agricultural production under stress conditions and improving the ecological environment.

**Fig. 1:**
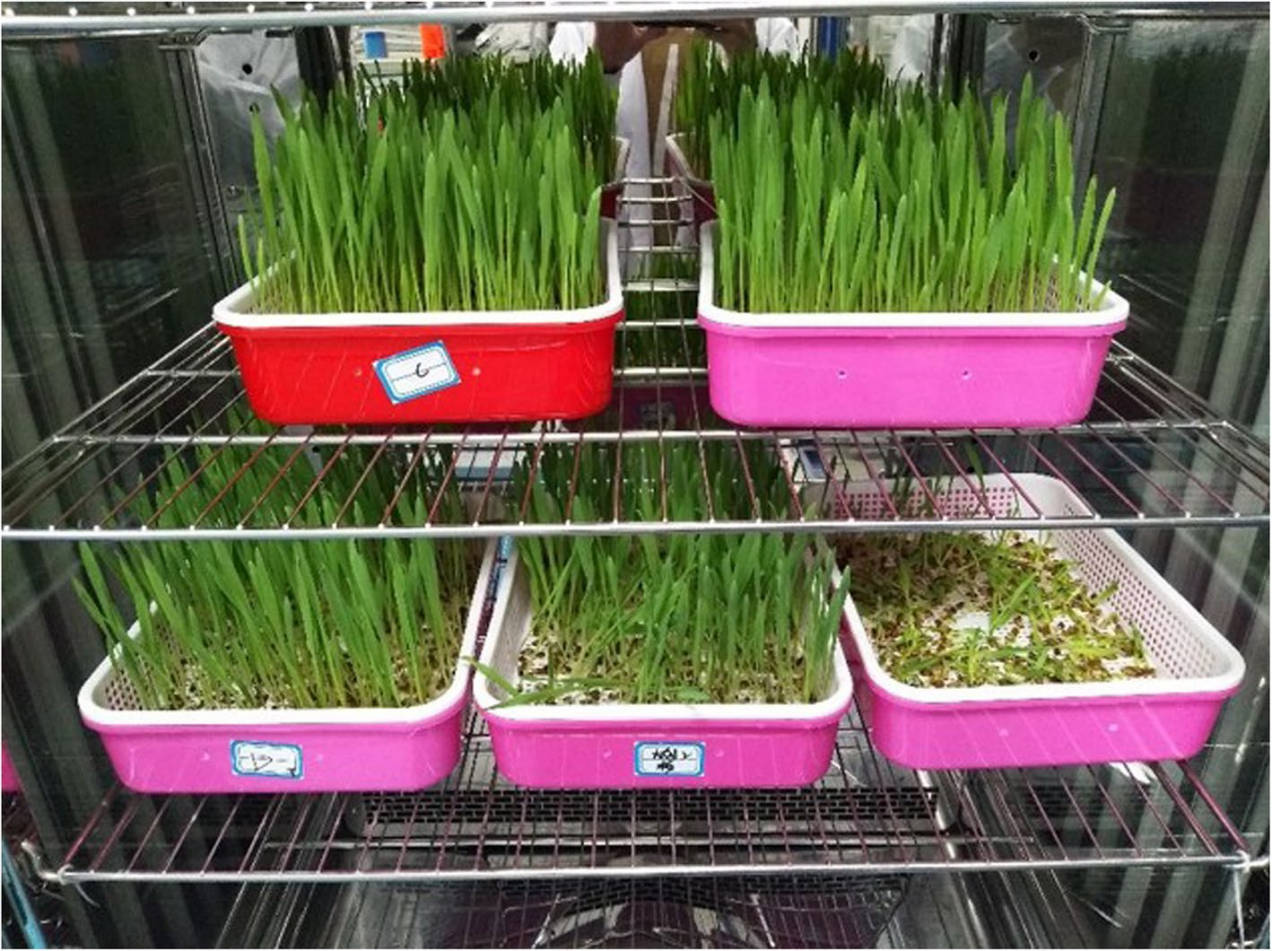
The highland barley in laboratory

In this study, through laboratory simulation of freeze-thaw, drought, and artemisinin stress conditions, highland barley was used as the experimental material (Fig. 1) to detect superoxide dismutase (SOD) activity, superoxide dismutase (POD) activity, and net photosynthetic rate, relative water content (RWC), malondialdehyde (MDA), relative electrical conductivity, content, soluble protein content response in highland barley, to analysis effects of freeze-thaw, drought, artemisinin stress on the physiological status of highland barley.

## Materials and methods

### Materials

In this paper, highland barley seeds of variety Beiqing No. 3 were obtained commercially in the market from an agricultural seed company Sichuan Tianlu Agricultural Science and Technology Development CO., LTD and used as the experimental material. The highland barley seeds were disinfected with 0.1% KMnO_4_ solution for 1 h and rinsed with deionized water. They were divided into 8 parts and numbered as no. 1-8, and distributed in plastic culture boxes. The plastic culture box could hold 500 mL Hoagland nutrient solution (Ca (NO_3_)_2_ = 0.945 g/L, KNO_3_ = 0.506 g/L, NH_4_NO_3_ = 0.080 g/L, KH_2_PO_4_ = 0.136 g/L, MgSO_4_ = 0.493 g/L). They were put in the SPX-300 light incubator for cultivation, where the temperature was set at 25 °C, 12 h light per day, and the culture was conducted for 6d. During this period, Hoagland’s nutrient solution was poured every day to ensure its normal growth.

## Methods

### Artemisinin, drought and freeze-thaw treatments

No. 1 ~ 4 were cultured with Hoagland’s nutrient solution. No. 5 ~ 8 were cultured with Hoagland’s nutrient solution with 20 mg/L artemisinin to simulate the allelopathy of artemisinin. All of them were put in the SPX-300 light incubator for cultivation, where the temperature was set at 25 °C, 12 h light per day, and the culture was conducted for 6d.

On day 6, No. 3 and 4 were cultured with Hoagland’s nutrient solution with 100 g/L polyethylene glycol, to simulate drought stress [23]. No. 7 and 8 were cultured with Hoagland’s nutrient solution containing 20 mg/L artemisinin and 100 g/L polyethylene glycol, to simulation artemisinin allelopathic effects and drought stress. The other groups did not change.

After 8 days of treatment under the above conditions, the No. 2, 4, 6 and 8 were put into BPHJ-120A high–low-temperature test chamber to carry out a freeze–thaw cycle for a period of 14 h, with the constant temperature curve set at 10, − 5, and 10 °C (T1, T2, and T3), while the others were maintained in the SPX-300 light incubator. The initial setting temperature of the BPHJ-120A high–low-temperature test chamber was 15 °C which was close to room temperature at night. The temperature dropped steadily to − 5 °C at a speed of 5 °C per 2 h and then the temperature rose from − 5 to 10 °C at a speed of 5 °C per 2 h. In each sampling time, samples of 8 treatment groups were randomly selected. Three replicate samples were wrapped in tin foil and frozen in liquid nitrogen, and then stored in an ultra-low temperature refrigerator at − 80 °C.

### Determination of indicators

#### SOD activity and POD activity

The SOD activity and POD activity were measured with the enzyme kit produced by the Institute of Nanjing Jiancheng Biological Engineering, for which 0.25 g leaf tissue for different treatments were ground with 5 mL phosphate buffer in an ice-water bath and centrifuged at 2500 r min − 1 for 10 min.

#### Relative water content (RWC)

The oven-drying method was used to determine the water content of leaves RWC. Zero point one gram of fresh leaf samples were taken, and the fresh weight (Wf) was measured. Immersing the leaf samples were done in water for 24 h. After drying the surface moisture of the samples with absorbent paper, the saturated weight (Wt) was measured. Finally, the leaf samples were baked to a constant weight at 120 °C, and the dry weight (Wd) was measured.

The RWC was calculated with the following Eq.

$$\mathrm{RWC}=\frac{\mathrm{Wf}-\mathrm{Wd}}{\mathrm{Wt}-\mathrm{Wd}}\times 100\%$$

#### Relative electrical conductivity

Plant leaves of the same size (as far as possible to ensure the integrity of the leaves, less stem nodes) were taken and rinsed with distilled water 3 times. Absorbed the surface water with filter paper, and cut the leaves into long strips of suitable length (avoid opening the main vein), quickly weighed 5 fresh samples, each 0.1 g, and placed them in a graduated test tube of 10 mL deionized water, covered with a glass stopper and soaked for 6 h at room temperature. Measured the extraction with a conductivity meter Liquid conductance (R1), then heated in a boiling water bath for 40 min, cooled to room temperature and shaken, then measured the extractant conductance (R2).

$$\mathrm{Relative}\ \mathrm{electrical}\ \mathrm{conductivity}=\mathrm{R}1/\mathrm{R}2\times 100\%.$$

#### Malondialdehyde (MDA) content

Thiobarbituric acid (TBA) method was used to determine malondialdehyde content. Zero point five gram of the samples were weighed, 1 ml of 10% trichloroacetic acid (TCA) was added and ground to homogenate, then 4 ml of 10% TCA was added and further ground to homogenate, and the supernatant was taken after centrifugation at a speed of 4000 r/min for 10 min. After centrifugation, 2.0 ml of the supernatant (control group with 2.0 ml distilled water) was absorbed in the test tube, and then 2.0 ml of 0.6% TBA solution was added. After mixing, the test tube was placed in boiling water bath for reaction for 15 min. After removal, the samples were rapidly cooled and centrifuged. The absorbance under 450, 532, and 600 nm was measured at last.

$$\mathrm{cMDA}=6.45\times \left(\mathrm{D}532-\mathrm{D}600\right)-0.56\times \mathrm{D}450$$

$$\mathrm{MDA}=\frac{\mathrm{cMDA}\times {\mathrm{V}}_{\mathrm{T}}}{{\mathrm{F}}_{\mathrm{W}}}$$

where V_T_ is the volume of extracted liquid (ml); F_W_ is the fresh weight of leaves (g).

#### Net photosynthetic rate

Put the sample in the incubator to recover for 30 min, and measure it with the CIRAS-3 portable photosynthesis system.

#### Soluble protein content

Coomassie brilliant blue G-250 staining (Zou 2000) was used. Centrifugal supernatant was taken firstly. After extracting 1.0 ml sample and fivefold dilution (4 ml distilled water was added) and then taking 1.0 ml to a test tube, each sample was repeated twice, adding 5 ml Coomassie brilliant blue G-250 solution and shaking. After 2-min accession, at 595 nm, the absorbance was measured by the standard curve.

### Data processing

The experimental data were graphed with Origin Pro9.0, and statistical analysis was performed with R 3.3.1 statistical software (R Foundation for Statistical Computing, Vienna, Austria). The t-tests were used to determine whether there were significant differences between groups. And we made the Pearson correlation analysis.

## Results

### The response of the net photosynthetic rate of highland barley to freeze-thaw, drought and artemisinin stress

**Fig. 2:**
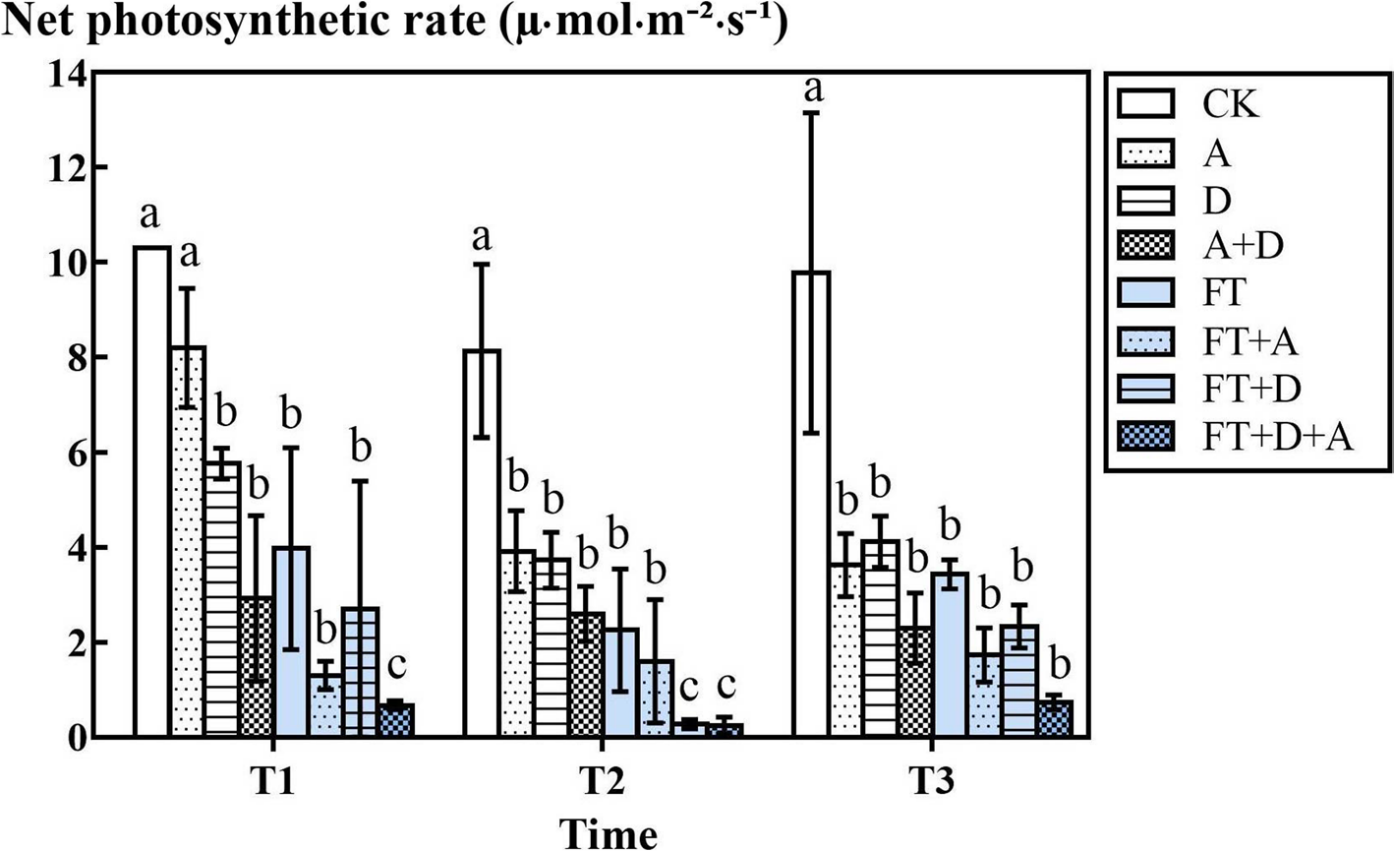
The effects of freeze-thaw (FT), drought(D) and artemisinin(A) stress on the net photosynthetic rate of highland barley. In Figures, T1 represents the temperature dropping to 10 °C after freeze-thaw for 2 h; T2 represents the temperature dropping to − 5 °C after freeze-thaw for 8 h; T3 represents the temperature rising back to 10 °C after freeze-thaw for 14 h

The Fig. 2 shows the response of the net photosynthetic rate of highland barley to freeze-thaw, drought and artemisinin stress. In the freeze-thaw groups, the net photosynthetic rate of the D, A, and D + A groups was significantly lower than that of the CK group (*P* < 0.05), respectively, with an average reduction of 44.7, 52.1, and 72.3%. Although the net photosynthetic rate of the D + A group was lower than that of the D and A groups, the difference was not significant (*P* > 0.05). In the freeze-thaw group, the net photosynthetic rate of the FT1, FT2, and FT3 groups were reduced by 61.2, 71.6, and 63.8% respectively compared with the CK group (*P* < 0.05). The FT + (D, A, D + A) groups decreased, compared with the FT groups. The lowest value of the net photosynthetic rate appeared in the FT2 + D + A group.

Net photosynthetic rate reflects the rate at which plants accumulate organic matter through photosynthesis. Compared with the CK group, the decrease of the net photosynthetic rate of the A group was due to the allelopathy of artemisinin which inhibited photosynthesis, and the reason may be that artemisinin inhibited the synthesis of chlorophyll in highland barley [24]. The net photosynthetic rate of the D and D + A groups was lower than the CK group’s, and it was probably because that the drought stress decreased the contents of the chlorophyll a, b, and total chlorophyll [25, 26]. The FT1 ~ 3 groups decreased by 61, 72, and 65%, respectively, indicating that low temperature had a stronger effect on plant photosynthesis, especially sub-zero temperature. This may be because low temperature inhibited the electron transport of thylakoids [27] and the activity of enzymes related to the Calvin cycle [28]. Under dual stress conditions, the net photosynthetic rate was lower than that of single stress, that is, compound stress showed the result of single stress superimposition. Under triple stress conditions, the net photosynthetic rate of barley was 0.7, 0.3, and 0.7 respectively, which were close to 0, that is, barley barely survived under such stress conditions and basically stopped growing. The experimental results are consistent with the research on bananas by Q. ZHANG, J.Z. ZHANG and others [29].

### The response of the SOD activity of highland barley to freeze-thaw, drought and artemisinin stress

**Fig. 3:**
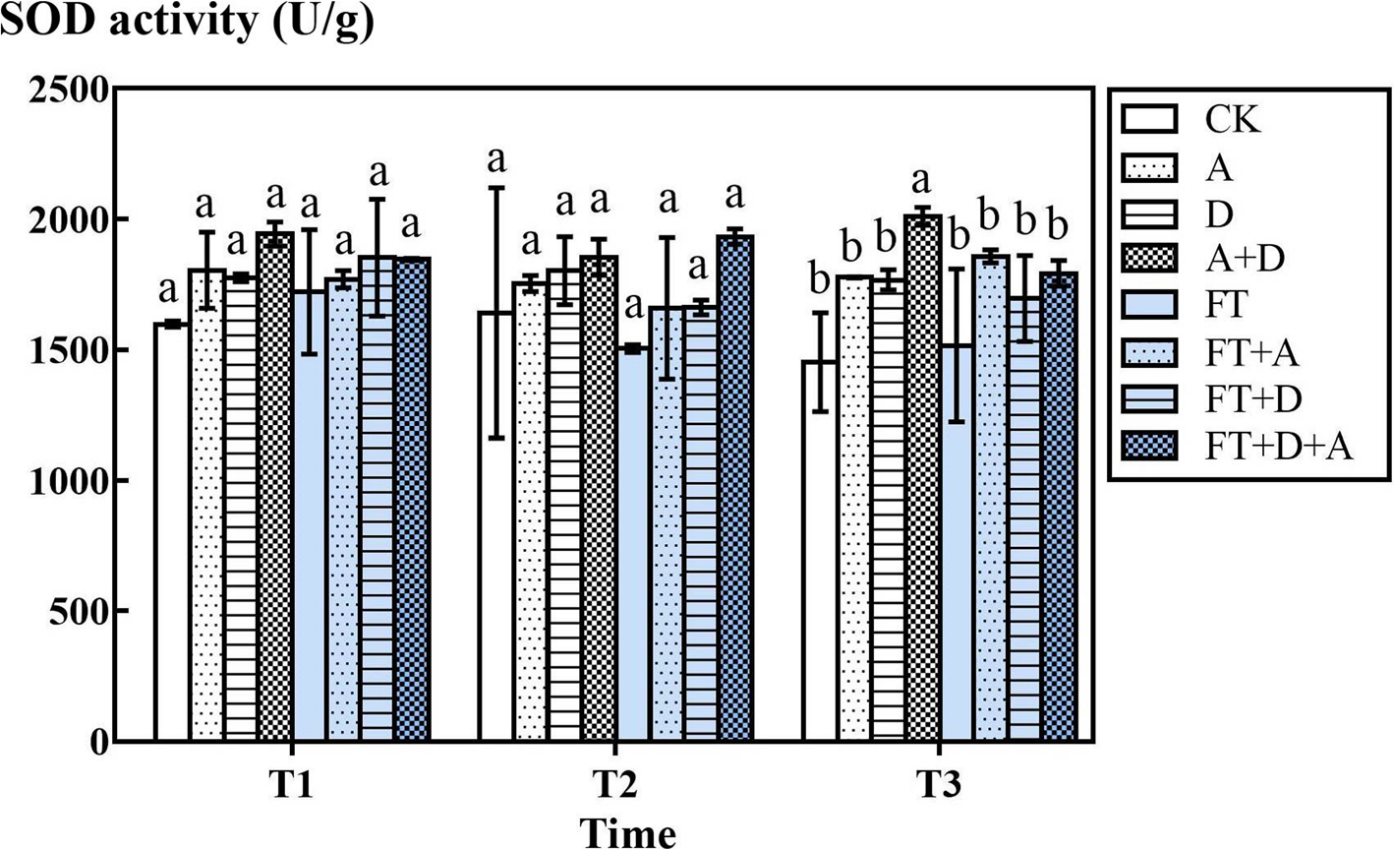
The effects of freeze-thaw (FT), drought(D) and artemisinin(A) stress on the SOD activity of highland barley. In Figures, T1 represents the temperature dropping to 10 °C after freeze-thaw for 2 h; T2 represents the temperature dropping to − 5 °C after freeze-thaw for 8 h; T3 represents the temperature rising back to 10 °C after freeze-thaw for 14 h

Figure 3 shows that SOD activity increased under stress conditions, but compared with CK group, single-factor stress such as A group, D group, and FT groups had no obvious impact (*P* > 0.05); while multi-factor stress (A + D group, FT2 + D + A group, FT3 + A group) had a significant impact on SOD (*P* < 0.05).

According to the study of Nievola et al., there are two main strategies for plants to cope with environmental stress: avoidance that prevents the stress factors from modifying plant functions, and tolerance in which physiological mechanisms are activated or modified to either resist stress or repair the damage [30]. As one of the main antioxidant enzymes, SOD is the first line of defense to protect plant cells from free radical damage [31]. When plants are subjected to adversity, a large amount of reactive oxygen species will accumulate in tissue cells, leading to lipid membrane peroxidation and inducing programmed cell death [32]. SOD is the only enzyme that uses free radicals as a substrate in the organism. It can scavenge superoxide anion free radicals to protect cells from damage [33], and plays an important role in the balance of oxidation and antioxidation [34, 35]. In addition, studies have shown that the antioxidant enzyme trend increased at first day of stress but did not continue and then increased again after a few days —— the regulation of the antioxidant enzyme was recidivous [36].

In this paper, SOD activity was expressed by the SOD activity per gram fresh tissue sample, while POD activity was expressed by the POD activity per gram protein. Under the action of single stress, the activity of SOD was higher than that of CK group, and double stress was higher than single stress, but under the action of triple stress, the SOD activity decreased. This showed that the allelopathy of artemisinin would damage plant cells and enhance the SOD activity. But the effect of freeze-thaw was even greater, because temperature and moisture changed strongly during the freeze-thaw process, and plants were synthesized through the synthesis of antioxidant enzymes. Through the synthesis of antioxidant enzymes, plants could eliminate the excess superoxide anion free radicals, reduce the excessive damage to plant tissues, and stabilize and maintain the structure and function of macromolecular substances in cells. Both stress increased the damage to plants. The SOD activity secreted by plants increased, so as to balance the body’s oxidation and anti-oxidation. Relevant studies have shown that the expression of antioxidant enzyme genes increases under stress conditions, which may be one of the reasons for the increasing of the SOD activity [37, 38]. However, the SOD activity decreased under triple stress. It indicated that when highland barley was subjected to three kinds of stress at the same time, the function of SOD in the leaves to scavenge free radicals was weakened, so that a large number of toxic substances accumulated in leaves, leading to damage to the leaves, until the plants stopped growing, or even died.

### The response of the relative water content of highland barley to freeze-thaw, drought and artemisinin stress

**Fig. 4:**
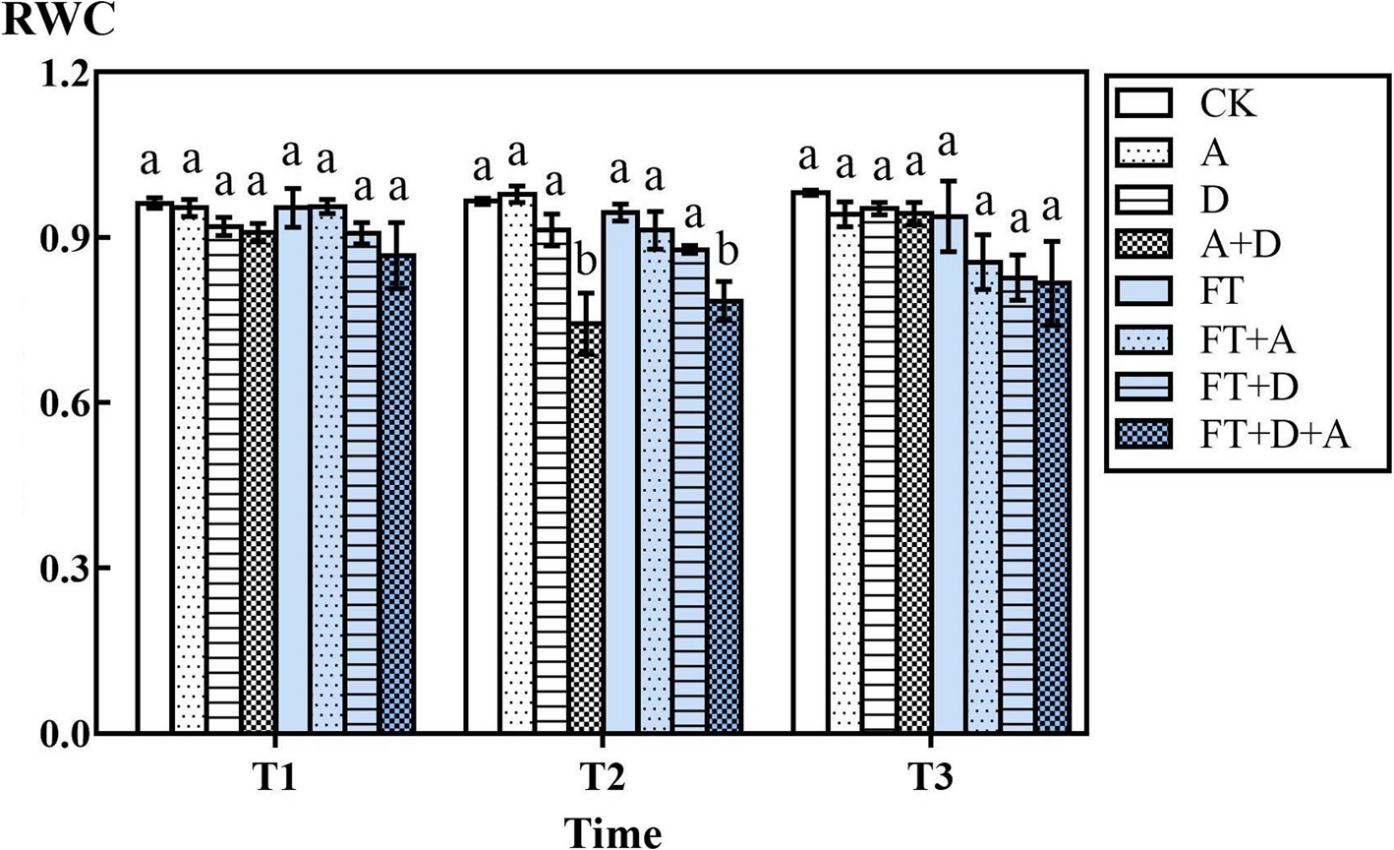
The effects of freeze-thaw (FT), drought(D) and artemisinin(A) stress on the relative water content of highland barley. In Figures, T1 represents the temperature dropping to 10 °C after freeze-thaw for 2 h; T2 represents the temperature dropping to − 5 °C after freeze-thaw for 8 h; T3 represents the temperature rising back to 10 °C after freeze-thaw for 14 h

It could be seen from the figure that the relative water content (RWC) decreased under stress conditions (Fig. 4), and the amount of decrease decreased with the increase of stress factors. However, compared with CK group, there were only two-factor and three-factor stress (D + A group, FT + A group, FT + D group and FT + D + A group) decreased significantly (*P* < 0.05), with an average decrease of 10.57, 6.44, 10.24, and 15.36% respectively.

The RWC reflects the degree of water deficit of plants under stress [39]. Water is an important part of plant growth and metabolism, so the change of leaf water under adversity is an important factor for evaluating drought tolerance and cold tolerance of plants [40]. Comparing the freeze-thaw stress groups and the control groups, the freeze-thaw stress would reduce the RWC of the leaves. Studies have shown that at low temperatures, plants reduced the RWC and increased the cell sap concentration to reduce the possibility of cell sap freezing [41]. From T2 to T3, the RWC of leaves continued to decrease for a period of time. It may be because as the temperature rose again, the normal physiological metabolic functions of plants would be destroyed owing to the rapid melting of water, which showed that the thawing process would also cause damage to plants [42]. The decrease in RWC in D group indicated that drought stress caused water deficit and reduced the RWC of plant leaves, which is consistent with the research of Marina Medeiros de Araújo Silva [43]. The RWC of cells also decreased under the condition of artemisinin stress, which may be because the allelopathy of artemisinin inhibited water absorption and storage.

### The response of the relative electrical conductivity of highland barley to freeze-thaw, drought and artemisinin stress

**Fig. 5:**
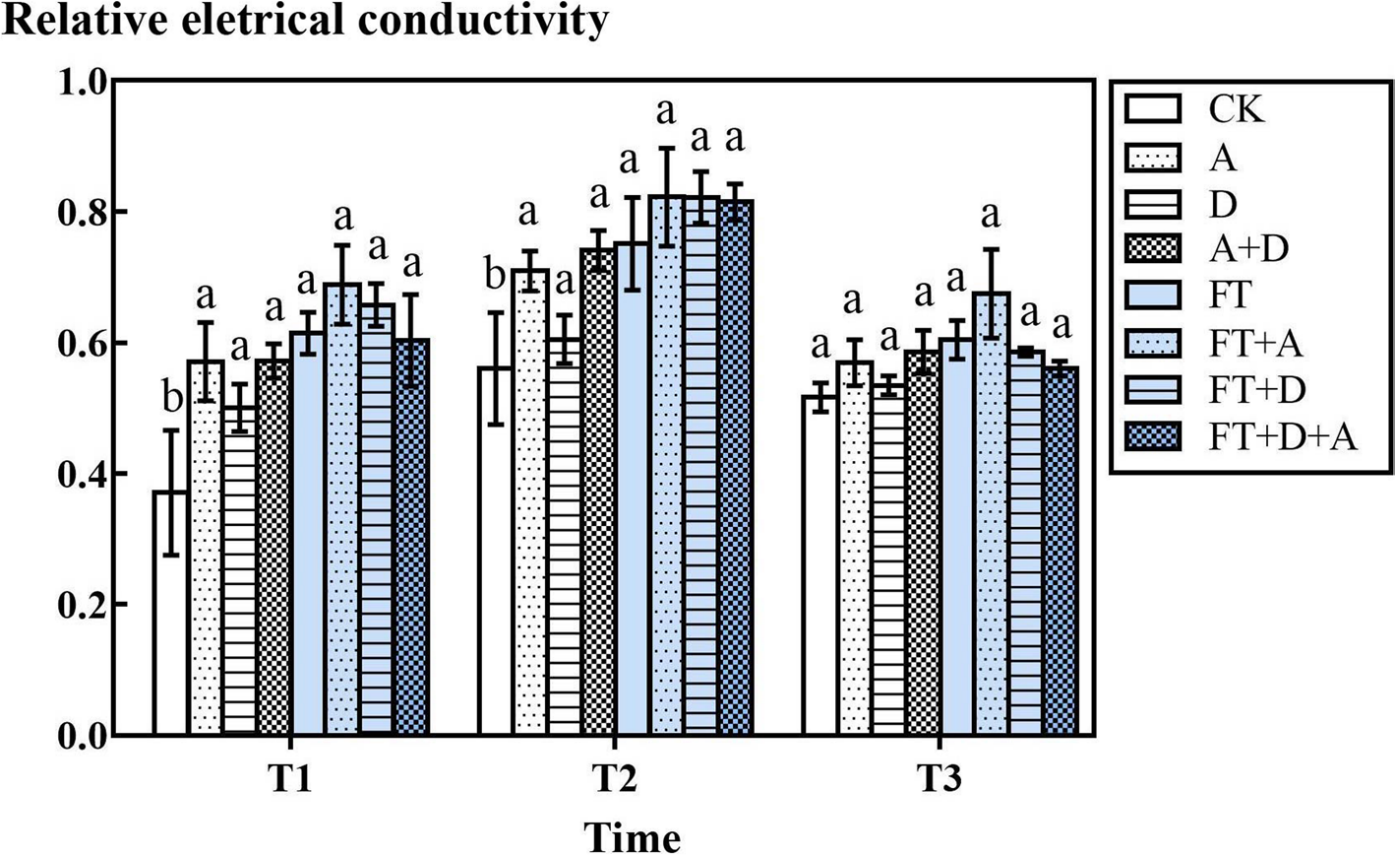
The effects of freeze-thaw (FT), drought (D) and artemisinin (A) stress on the relative electrical conductivity of highland barley. In Figures, T1 represents the temperature dropping to 10 °C after freeze-thaw for 2 h; T2 represents the temperature dropping to − 5 °C after freeze-thaw for 8 h; T3 represents the temperature rising back to 10 °C after freeze-thaw for 14 h

According to Fig. 5, in the non-freeze-thaw groups, the relative electrical conductivity of the artemisinin-treated groups (A group, D + A group) was markedly higher than that of the CK group (*P* < 0.05). Compared with the CK group, the FT group had a significant increase, while the relative electrical conductivity of the FT group at T2 was significantly greater than that at T1 (*P* < 0.05).

The cell membrane permeability under adversity stress is one of the important indicators to measure plant stress tolerance. The plasma membrane permeability directly reflects the degree of injury to the plant plasma membrane system. The plasma membrane permeability can be measured by the extravasation rate of plant cell fluid, that is, the relative electrical conductivity.

Compared with the CK group, the increase in the relative electrical conductivity of the A and D groups indicated that the allelopathy of artemisinin and drought stress could damage the plant plasma membrane system and increase the plant’s plasma membrane permeability. Some research shows that oxidative injury could manifest at the cellular membrane by accumulation of MDA, ultimately leading to increase in electrolyte leakage in freeze-thaw tissues [44, 45]. Compared with the CK group and the FT group, it was found that low temperature had a more obvious effect on the relative electrical conductivity of plants, especially at T2 (− 5 °C). Plants resist ice formation at freezing temperatures by the generation of high levels of osmolytic solutes to maintain normal hydration and prevent the formation of ice crystals [46], which may be the reason of the increasing of the relative electrical conductivity of plants. The result is consistent with the research of Hajihashemi [47]. Composite stress would further increase the relative electrical conductivity of barley, which was the result of the superimposed effects of single factors.

### The responses of MDA content of highland barley to freeze-thaw, drought and artemisinin stress

**Fig. 6:**
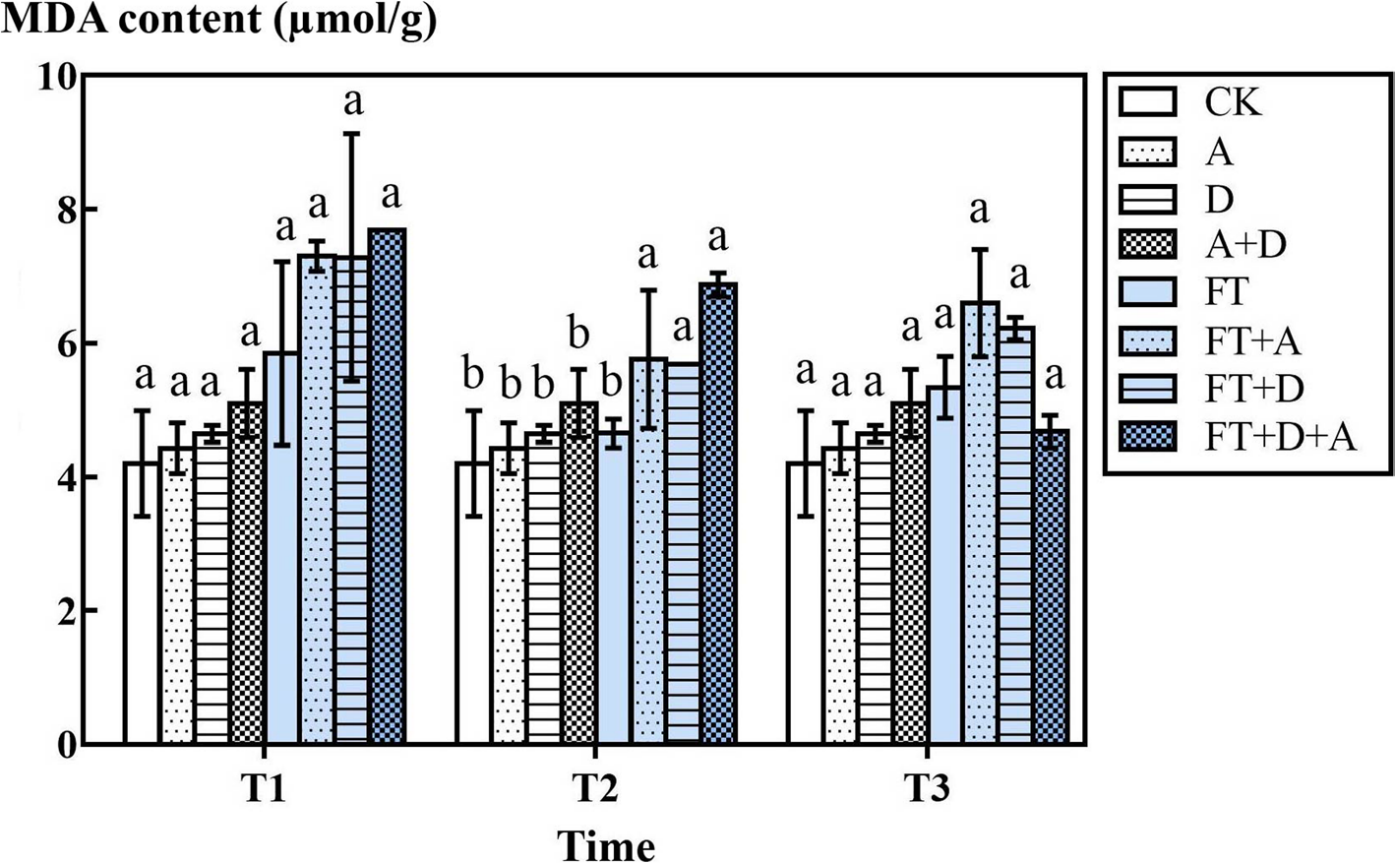
The effects of freeze-thaw (FT), drought(D) and artemisinin(A) stress on MDA content of highland barley. In Figures, T1 represents the temperature dropping to 10 °C after freeze-thaw for 2 h; T2 represents the temperature dropping to − 5 °C after freeze-thaw for 8 h; T3 represents the temperature rising back to 10 °C after freeze-thaw for 14 h

According to Fig. 6, in the non-freeze-thaw groups, the MDA content of highland barley increased in the D, A, and D + A groups compared with the CK group, but it was not significant (*P* > 0.05). In the freeze-thaw groups, the MDA content of highland barley increased markedly in the multi-factor stress group—FT + A group, FT + D group, FT + A + D group (except FT + A + D group at T3 temperature) compared with the CK group (*P* < 0.05).

The content of MDA is an important parameter that reflects the body’s anti-oxidation potential. MDA content not only reflects the rate and intensity of lipid peroxidation in the body, but also indirectly reflects the degree of tissue peroxidation damage, which is a commonly used indicator in the research of plant senescence physiology and resistance physiology. Membrane lipid peroxidation often occurs when plant organs age or suffer damage under adversity. MDA is the final decomposition product of membrane lipid peroxidation, and its content can reflect the degree of damage to plants under adversity [48, 49].

Under freeze-thaw and compound stress, the content of MDA in the barley leaves increased due to the damage of the highland barley membrane caused by the stress factors. When the experimental temperature dropped from T1 (10°C) to T2 (− 5 °C), it was found that the MDA content in the barley leaves decreased, indicating that the lipid peroxidation reaction in the body decreased. This was because the highland barley, as a major crop in the alpine regions of China, has a certain degree of cold resistance [50], and can synthesis of more antioxidant enzymes to reduce oxygen-containing free radicals, thereby reducing cell membrane damage caused by environmental stress leads to a decrease in MDA concentration [47]. When the temperature increased to T3 (10°C), the membrane lipid peroxidation in the barley leaves increased, and the MDA content increased, but under this condition MDA was not higher than the temperature of T1 (10°C). At T3, the MDA content of barley in the FT + A + D group was different from that of T1 and T2, but was markedly lower than that in the FT group, FT + A group and FT + D group. This may be because the leaves were severely damaged under a freeze-thaw cycle under multiple stress, resulting in physiological abnormalities.

### The response of the soluble protein content of highland barley to freeze-thaw, drought and artemisinin stress

**Fig. 7:**
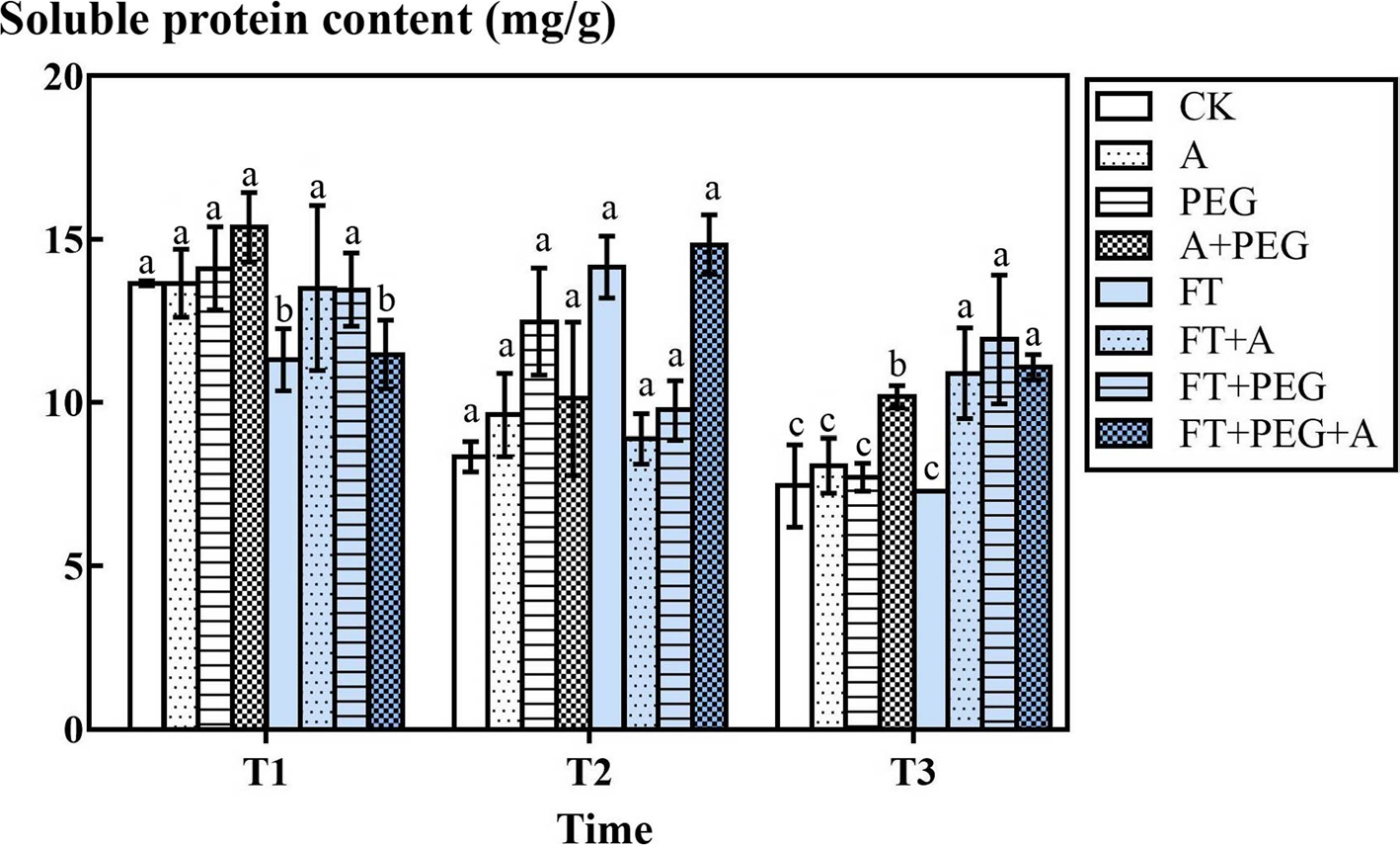
The effect of freeze-thaw (FT), drought(D) and artemisinin(A) stress on the soluble protein content of highland barley. In Figures, T1 represents the temperature dropping to 10 °C after freeze-thaw for 2 h; T2 represents the temperature dropping to − 5 °C after freeze-thaw for 8 h; T3 represents the temperature rising back to 10 °C after freeze-thaw for 14 h

According to Fig. 7, in the non-freeze-thaw groups, compared with the CK group, the D, A, and D + A groups increased slightly, but not significantly (*P* > 0.05). Only the D + A group of T3 increased markedly (*P* < 0.05). In the freeze-thaw groups, the FT groups and the FT + A + D group decreased significantly at T1 temperature (*P* < 0.05), and compared with the non-freeze-thaw groups, the FT + A group and FT + D group had no significant changes; however, the T2 temperature was different from that of the non-freeze-thaw groups. The FT groups and the FT + A + D group increased significantly (*P* < 0.05), but the FT + A group and FT + D group had no significant changes compared with the non-freeze-thaw groups. For T3 temperature, the FT + A group, FT + D group and FT3 + A + D group were markedly higher than CK group (*P* < 0.05).

Soluble protein is an osmotic adjustment substance of plant cells. High content of soluble protein can maintain the osmotic pressure of plant cells, thereby resisting the damage caused by drought and low temperature [51]. According to Pu’s study, in the freezing stress, seedling dehydration, chloroplast dilation and degradation, an increase in the content of MDA, proline, soluble protein and soluble sugars, as well as REL showed rapid growth [52], which is consistent with our result. The increase of soluble protein concentration in non-freeze-thaw groups verified this point. During the freeze-thaw process, the soluble protein content at T1 temperature decreased. It showed that the highland barley has not adapted to the adversity in the early stage of freeze-thaw. The cell membrane damage led to the loss of protein, and part of the protein was used for membrane repair. After that, the concentration of soluble protein at T2 and T3 increased, which demonstrated that the highland barley synthesized a large amount of new protein to maintain cell osmotic pressure, resist damage from external adversity, and show gradual adaptation to it. When the temperature increased, the pressure slowed down and the protein synthesis continued. This is consistent with the change of soluble protein content during freeze-thaw cycle of Bian et al. [53].

### The response of the POD activity of highland barley to freeze-thaw, drought and artemisinin stress

**Fig. 8:**
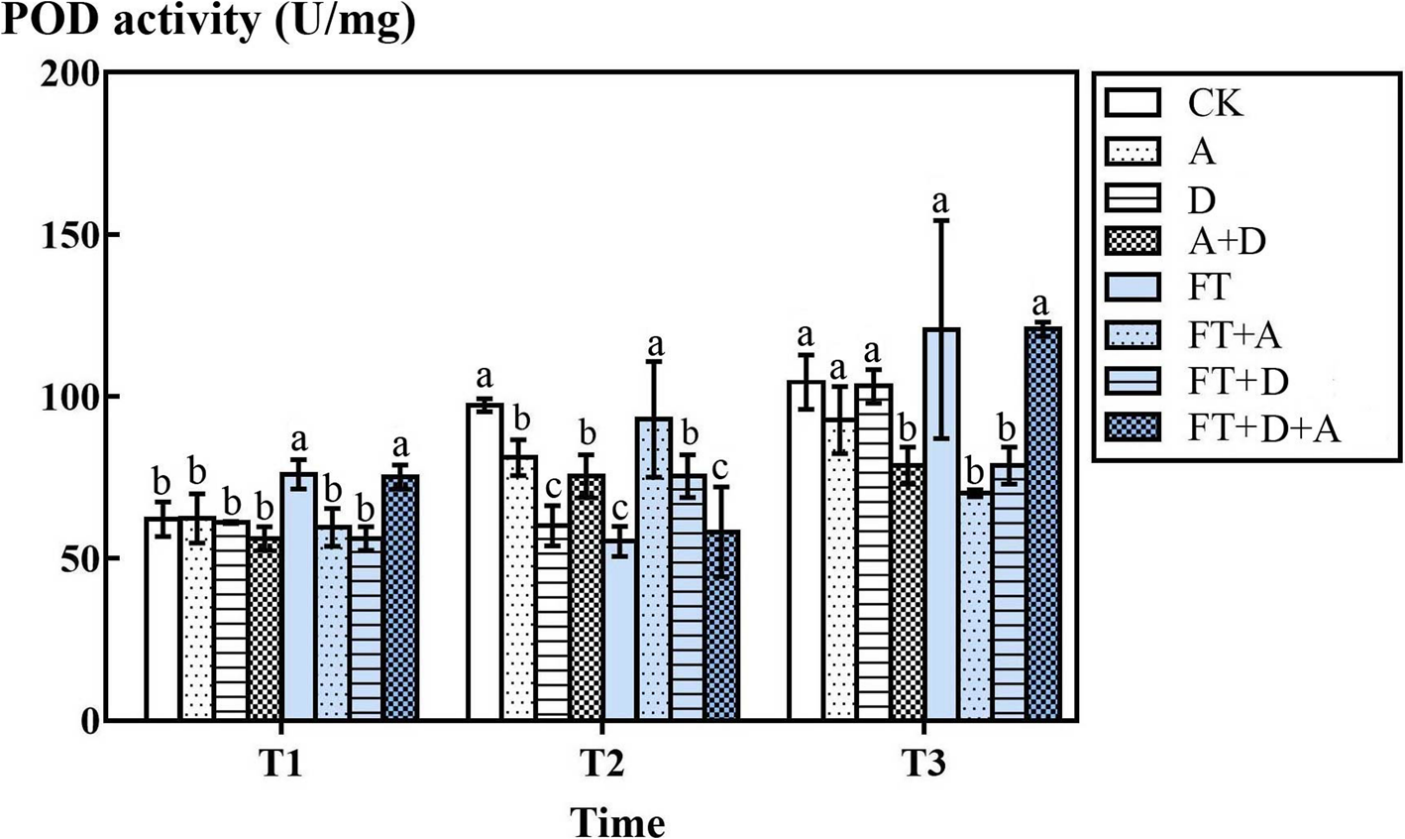
The effects of freeze-thaw, drought and artemisinin stress on the POD activity of highland barley. In Figures, T1 represents the temperature dropping to 10 °C after freeze-thaw for 2 h; T2 represents the temperature dropping to − 5 °C after freeze-thaw for 8 h; T3 represents the temperature rising back to 10 °C after freeze-thaw for 14 h

According to Fig. 8, in the non-freeze-thaw groups, compared with CK group, D group, A group and D + A group increased slightly, but not markedly (*P* > 0.05), only D + A group of T3 increased significantly (*P* < 0.05). In the freeze-thaw groups, for the low temperature above zero, the temperature of T1 and T3 in FT groups and FT + A + D groups increased markedly (*P* < 0.05) There was no significant change in FT + A group and FT + D group compared with non-freeze-thaw groups at T1 temperature, but there was a decrease in FT + A group and FT + D group compared with non-freeze-thaw groups at T3 temperature (*P* < 0.05). The T2 temperature of subzero low temperature was different from that of the non-freeze-thaw groups, FT group and FT + A + D group decreased significantly (*P* < 0.05), but compared with non-freeze-thaw groups, FT + A group and FT + D group increased significantly (*P* < 0.05).

POD widely presents in different tissues of plants. It can remove peroxides in the body under adversity and protect plants from active oxygen damage [54]. Different from SOD activity, POD activity was expressed by the POD activity per gram protein in this paper. When temperature was above zero, the POD activity in the leaves of highland barley increased under freeze-thaw and combined stress, which also verified this conclusion. There was a significant negative correlation between POD activity and MDA content (Table 1), which also showed that POD could scavenge reactive oxygen species to protect the membrane. In the non-freeze-thaw groups, the POD activity of D group, A group, D + A group at T2 and the D + A group at T3 were significantly lower than that of CK group. At T3 temperature, the POD activity of FT + A group and FT + D group decreased compared with non-freeze-thaw groups. Sheteiwy’s research was different from the results of this paper: the antioxidant enzymes such as POD and CAT in soybeans were highly activated under abiotic stresses to adapt, control, and to scavenge the free radicals induced by drought stress [55]. According to the extremely significant negative correlation between POD activity and soluble protein (Table 1), it could be inferred that the high concentration of soluble protein led to the decrease of POD activity per unit protein, but further research is needed to explain this phenomenon.

**Table 1:** Pearson correlation analysis of POD activity, SOD activity, net photosynthetic rate (Pn), RWC, relative electrical conductivity (REC), MDA content, and soluble protein under freeze-thaw, drought, and artemisinin stress

	POD	SOD	Pn	RWC	REC	MDA	Soluble protein
POD	1	−0.38	0.173	0.066	−0.216	−0.516*	− 0.867**
SOD		1	−0.126	−0.067	− 0.055	0.413	0.288
Pn			1	−0.222	−0.650**	− 0.498*	0.096
RWC				1	−0.225	0.111	−0.029
REC					1	0.243	−0.035
MDA						1	0.243
Soluble protein							1

### Pearson correlation analysis of indicators

The Table 1 is the correlation coefficient matrix of seven indexes under FT + D + A conditions. The “*” represents significant correlation (*P* < 0.05), and “**” represents extremely significant correlation (*P* < 0.01). It was found that POD activity and MDA content, net photosynthetic rate and MDA content were significantly correlated, and POD activity and soluble protein content, net photosynthetic rate and relative electrical conductivity are extremely significantly correlated.

## Conclusion

Under the conditions of freeze-thaw, drought, artemisinin and their combined stresses, the physiological status of barley had undergone a series of changes. Under stress, the concentration of soluble protein in the highland barley, as an osmotic adjustment substance in cells, increased, the relative water content of the cells decreased, and the relative conductivity increased to increase the water retention capacity of the cells and resist adversity. The increase of MDA concentration indicated that the membrane in the cell was damaged, while the highland barley increased the SOD activity to resist stress conditions and reduce cell damage. Both drought and artemisinin could cause the above effects, but compared with that, the combined stress of drought and artemisinin was, in most cases, not significant, from which it could be inferred that drought stress and artemisinin stress showed a certain degree of antagonism. The net photosynthetic rate of highland barley was more obviously affected by stress, and photosynthesis was inhibited in both single stress and compound stress. Under stress conditions, the POD activity decreased, which might be obscured the effect (due to the strong change of soluble protein). In the freeze-thaw cycle, low temperature had the above adverse effects on highland barley. However, the net photosynthetic rate, relative water content and SOD activity had no significant differences among the three temperatures. MDA content increased most obviously at the initial stage of freeze-thaw cycle, while the response of the concentration of soluble protein and relative electrical conductivity was most obvious at the freezing stage. These results indicated that the osmotic regulation of highland barley cells was the most significant under freezing condition, and the cells had lipid damage under low temperature but could gradually reduce the damage and exhibit a certain adaptability. This research was conducted in a laboratory with artificial stress simulation, and outdoor experiments can be considered in the future.

## Data Availability

All data generated or analyzed during this study are included in this published article.
